# Adoption of Blockchain in Health Care

**DOI:** 10.2196/17423

**Published:** 2020-09-17

**Authors:** Mark Gaynor, Janet Tuttle-Newhall, Jessica Parker, Arti Patel, Clare Tang

**Affiliations:** 1 School of Public Health and Social Justice Saint Louis University St Louis, MO United States; 2 Brody School of Medicine at East Carolina University Greenville, NC United States

**Keywords:** blockchain adoption, blockchain technology in health care, supply chain, data management

## Abstract

This study aims to review current issues regarding the application of blockchain technology in health care. We illustrated the various ways in which blockchain can solve current health care issues in three main arenas: data exchange, contracts, and supply chain management. This paper presents several current and projected uses of blockchain technology in the health care industry. We predicted which of these applications are likely to be adopted quickly and provided a supply chain example of tracking the transportation of organs for transplantation.

## An Introduction to Blockchain Technology

Blockchain technology is a distributed database that records and stores transaction records. Specifically, blockchain is a record of peer-to-peer transactions built from linked transaction blocks that are immutable and shared within a network.[1]. A distribution ledger is “a type of database that is shared, replicated, and synchronized among the members of a network. The distribution ledger records the transactions, such as the exchange of assets or data, among the participants in the network” [2]. Distribution ledgers can be classified as public or private. A public distribution ledger is anonymous in the sense that each user has a copy of the ledger and participates in confirming transactions independently, whereas a private distribution ledger is not anonymous. A permissioned blockchain requires that individuals be given a copy of the ledger and permission from an organization that oversees the ledger to participate in confirming transactions. This technology allows organizations to manage privacy and Health Insurance Portability and Accountability Act concerns as most will be private and require permission.

Blockchain technology, which is currently revolutionizing industries globally, is well suited for identity management, transaction processing, record management, and public health surveillance. This disruptive technology is creating innovative solutions for complex issues in a variety of industries including fishing, diamond, fashion, shipping, banking, and now health care, which are discussed in Multimedia Appendix 1 [3-11]. This paper discusses how this technology may solve some of the largest, most complex, and convoluted problems in the health care industry today. It also introduces a framework to assess the adoption of blockchain technology in health care proposed by Iansiti [12]. This framework is used to justify an example of blockchain application that is likely to be adopted quickly to a transportation chain of custody system for organs intended for transplantation.

**Figure 1:**
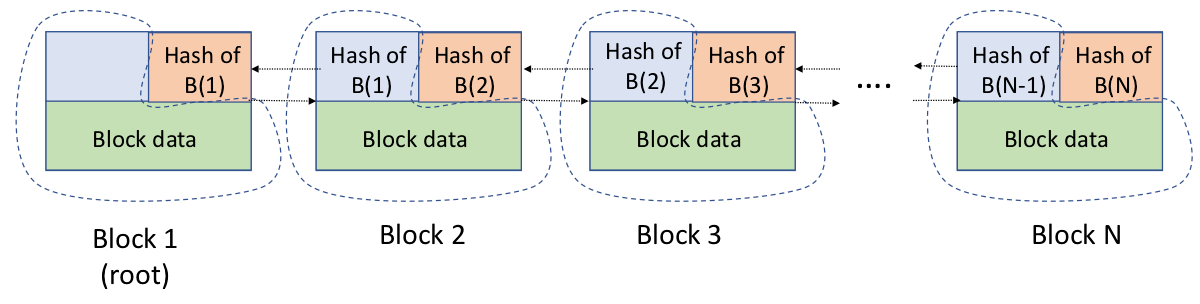
Simple blockchain.

Figure 1 illustrates a simple blockchain with N blocks. Each block has data, a hash of the previous block, and a hash of the data in this block. The dashed lines define the region where the block hash covers. Each block is linked to the previous block (except the root block) and every block in the secure chain. If any data in a block are changed, then the hash for that and later blocks will be incorrect. The distributed nature of blockchain means that everybody has a copy of the chain and must also make the same changes to keep the entire blockchain consistent, which is highly unlikely (ie, high Byzantine fault tolerance). This is a model of distributed trust where one or more *bad actors* do not compromise the integrity of the database.

Interactions among participants occur across the network to store, exchange, and view information. The ledger will permanently record data in a sequential chain where *confirmed and validated transaction blocks are linked and chained from the beginning of the chain to the most current block* [2]. Once a transaction is added to the block, it cannot be altered because each block must be verified from all users with access to the ledger, which ensures the integrity of the information being shared. As the blockchain network automatically conducts a self-check, it results in greater transparency and reduced corruptibility.

Blockchain technology does not use any form of centralized authority. The records are public and easily verifiable. In health care, these blocks will not be completely public, but those who are given permission will be granted access to verify whether the information is correct before it becomes part of the blockchain. An audit trail follows every transaction for authentication purposes, and each record has a timestamp and a unique cryptographic signature [2]. The cryptographic signature, also known as a digital signature, allows a user to sign with a private key to track the origination of the transaction information. Blockchain technology uses encryption for security control and authentication via a public and private key. A user’s public key is their address on the blockchain, whereas a user’s private key is similar to a password that provides access to data. The combination of public and private keys ensures that the data stored on the blockchain are incorruptible and traceable to the origin source while keeping that source anonymous.

A cryptographic hash serves as the digital signature for a block of data. Hashing, along with the use of public and private keys, proves that information in the transaction has not been altered. The goal of blockchain technology is to serve as a digital ledger that will eliminate the intermediaries with the use of cryptographic hashing and timestamps to establish digital trust among users and to allow direct and efficient transfer of data and information [13].

## Growing Interest in Blockchain and the Health Care Industry

On the basis of the successful use of blockchain in other industries, as described in Multimedia Appendix 1 [3-11], we examine and predict how health care could benefit from this technology and the speed of adoption of different applications in 3 arenas: data exchange, contracts, and supply chain management. Each arena is depicted below with an illustrated tree and description of the components making up each potential areas for improvement. These applications are derived from a set of relevant papers commissioned by the Office of the National Coordinator [1,2,14-18], other related research [19,20], and the authors’ experiences in the health care industry.

## Overview of Health Care Problems

Health care is a complex industry with various influential stakeholders. Blockchain-based technology has the capacity to disrupt the logistics of the health care industry through innovative solutions to the challenges faced in the industry, which include but are not limited to the following: (1) supply chain management, (2) technical issues in data management, (3) smart contracts, (4) confidentiality of personal health information (PHI), (5) enabling and/or assisting implementation and assessment of alternative payment models, and (6) virus outbreak tracking and surveillance.

### Data Exchange

Management of the large amount of data collected by health systems presents a technological challenge for health care systems, payors, regulatory agencies, government overseers, and professionals. Challenges in data management specifically include data structure, security, data standardization, data storage and transfers, governance and ownership of the data, inaccuracies, and real-time applicable analytics [21]. The two-part verification system utilized in blockchain technology creates potential solutions to these issues. The private or public key system in blockchain allows for trust to be established to efficiently transfer data in a manner that is traceable and secure.

**Figure 2:**
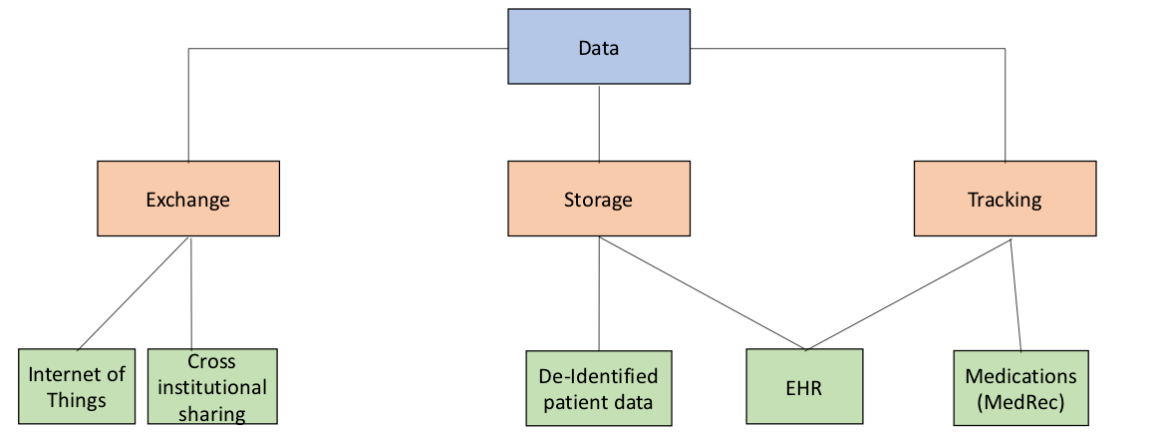
Data exchange tree. EHR: electronic health record.

Figure 2 graphically depicts some opportunities provided by blockchain for exchanging health care data. These applications could allow the health care industry to improve data exchange across all industry areas, including exchange, storage, and record tracking.

For long-term success, blockchain technology application must supplement the electronic health records (EHRs) that health care systems and providers are currently using. Certainly, for multiple reasons (ie, cost, compliance, and meaningful use requirements to name a few), blockchain technology cannot replace EHRs. Supply chain management for organ transport is a good example of an application where early adoption is likely because the application of this technology is well understood (not novel) and simple to implement (not complex) as it is independent of the EHR function. Blockchain technologies have already been developed on platforms such as Chronicled [22], which tracks a range of products including drugs, blood, and organs.

Likely applications to health care data management and storage include the following:

1. The potential to manage data created by a patient encounter with the Internet of Things (IoT) for cross-institutional data sharing. A blockchain infrastructure for the IoT could manage health data from wearable devices [15]. Blockchain could also be used for cross-institutional sharing of health care data via health information exchange networks, application programming interfaces, and the use of standards. Cross-institutional sharing will allow significant increases in clinical and research efficiency through access to data from multiple health care institutions and continuity of care across multiple health care venues based on provider access to consistent patient information.
2. Fast Healthcare Interoperability Resources (FHIR) and the smart security infrastructure ensure data privacy and interoperability with EHRs. These features allow individual institutions to retain operational control of their data and ensures that sensitive patient data are not shared on the blockchain for security and confidentiality measures [17]. Most importantly, FHIR allows for full collaboration among institutions and public health organizations for better care coordination, outcome-based care, population health as well as enabling other diverse data to be shared for research purposes.
3. Blockchain ensures privacy, security, and trust in real-time, distributed data structures. It also ensures provenance, data verification, and data accuracy. For example, linked hashes and public or private key cryptography will help ensure data integrity and tracking of ownership, which can be used to store parts of the EHR (or a pointer to it) on the blockchain to be accessed across multiple health care systems. A public blockchain could be used as an access control manager to health records that are stored off the blockchain. This will allow access to data through a secure user’s unique identifier, encrypted link to the health record, and timestamp of each transaction. Blockchain could serve as an immutable audit log where data queried on the blockchain are tracked to ensure that data are only accessed by authorized personnel [16].
4. Blockchain is also attempting to address the confidentiality of PHI through a secure and encrypted data exchange network [2]. Blockchain could enable patients to manage and control their own health information to stay educated and aware of their health care needs. One example is to allow patients to hold custody of their health care data and control where and how the data can be used to share health data in a timely manner for better patient care [21,23]. Strong encryption and an immutable transaction ledger will allow patients to use web-based or mobile apps to view and grant or revoke access to specific parties [23].
5. Finally, an MIT lab has developed a MedRec prototype that uses blockchain technology for data management and record keeping. MedRec manages authentication, confidentiality, accountability, and sharing of sensitive data while facilitating interoperability and making health information technology (IT) convenient and adaptable. Additionally, MedRec is integrated with the organization’s existing local data storage solutions to pull aggregated data across networks. “The MedRec prototype provides a proof-of-concept system, demonstrating how principles of decentralization and Blockchain architecture could contribute to secure, interoperable EHR systems” [15]. MedRec can also be used to track medications used by patients and serve as a repository for medication storage and management.
6. Now more than ever, blockchain technology can help monitor the spread of disease, patient data management, insurance, provider directories, and supply chain of food sources. As the Coronavirus disease 2019 pandemic continues to spread rapidly across the country, this technology can be used to trace cases and transmission patterns. Recently, the US Department of Homeland Security published guidelines for using blockchain to accurately track and trace the movement of goods in the supply chain [24]. IBM Food Trust is currently using distributed ledger technology (DLT) to monitor the movement of food throughout the United States to assess the food supply during this pandemic.

#### Contracts

**Figure 3:**
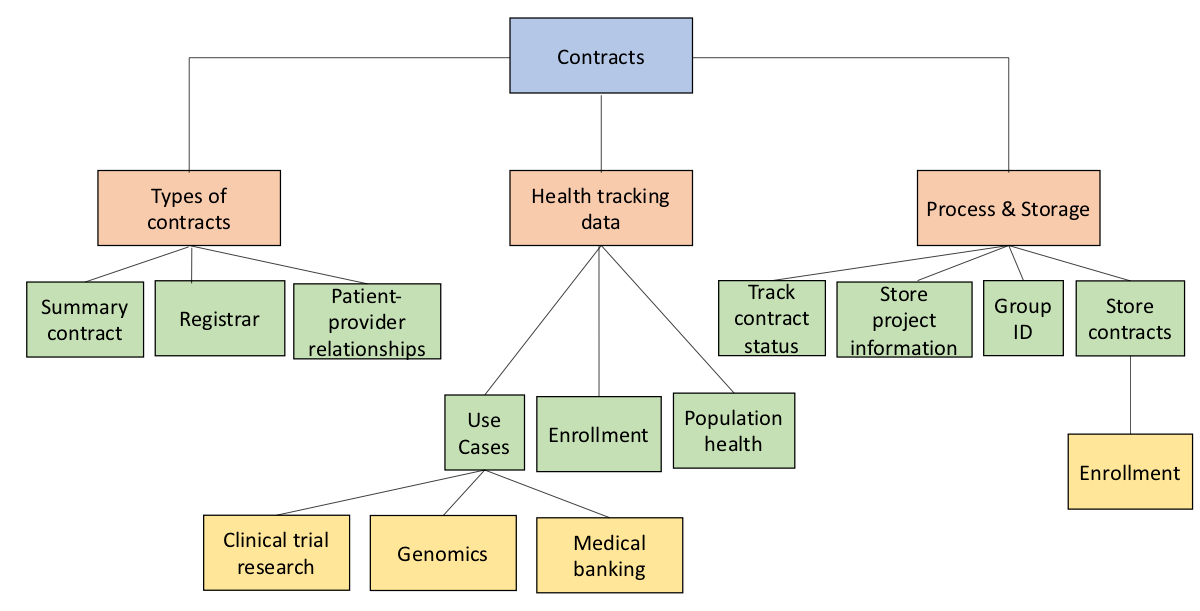
Smart contracts tree.

Figure 3 illustrates the opportunities where blockchain could be employed to improve contracts in the health care environment. Smart contracts allow users to automate and track certain state transitions. The party receiving new information receives an automated notification and can verify the proposed record before accepting or rejecting the data. This keeps participants informed and engaged in the evolution of the record. Overall, contracts are broken down into 3 branches: types of contracts available to use, health tracking data, and process and storage.

1. The first area of focus is the different types of smart contracts that allow users to automate and track state transitions. The party receiving new information receives an automated notification and can verify the proposed record before accepting or rejecting the data while keeping the participants informed. The 3 types of smart contracts are summary contracts, registrar contracts, and patient-provider relationship contracts [15]. A summary contract functions as a bread crumb trail for participants in the system to locate their medical record history. Providers have references to patients they serve and third parties with whom their patients have authorized data sharing [15]. Smart contracts allow participating parties to build a trusting relationship through increased transparency and tracking with each transition and record evolution.
2. Smart contracts could advance methods used for tracking health data. As previously mentioned, cross-institutional data exchange can allow data to be gathered on patients on a microscopic through to the macroscopic level when comparing patients with similar conditions and/or demographics [17]. Smart contracts could be used to track enrollment for health plans giving providers and patients a better understanding of benefit utilization [14]. In terms of population health, data gathered nationally could be accessed by researchers and institutions to study epidemiology when data (or pointers to these data) are stored in the blockchain, making the data interoperable, secure, and easily accessible and traceable. Smart contracts allow participating parties to understand the utilization of health data while providing secure and auditable tracking of access.
3. One significant benefit of a smart contract is its ability to provide process and storage for contracts, project management, and health data. Contracts and other vital information are stored on the blockchain, establishing a network of trust among the parties involved. This allows health organizations to share patient data that are deidentifiable, ensuring confidentiality. The decentralized database solution provided by blockchain is a key resolution to interoperability and record storage while maintaining health data and other health-related information [2].

#### Supply Chain

**Figure 4:**
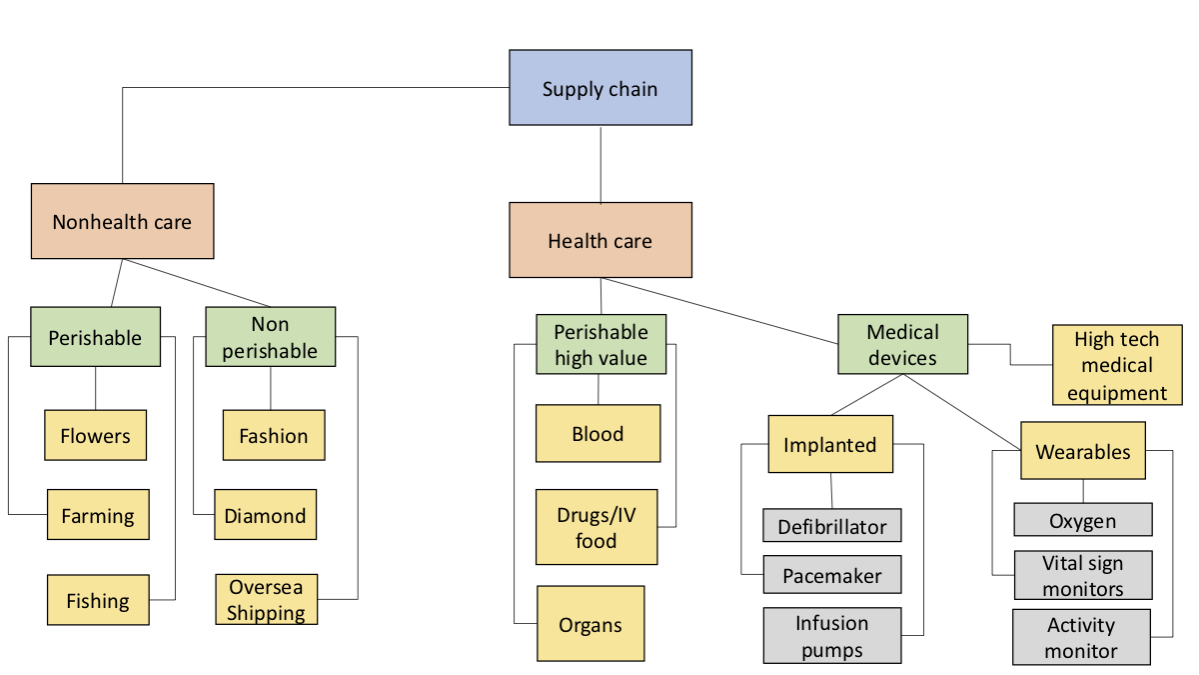
Supply chain tree. IV: intravenous.

In health care, issues arise with transparency and IT and tracking costs when following items along the supply chain [21]. Blockchain has the power to solve these issues by creating more transparent, instantaneous tracking of high-value items through its shared digital ledger. The final tree in Figure 4 illustrates the opportunities for the use of blockchain in the medical supply chain. These areas include high-value items, medical devices, and durable medical equipment.

1. Supply chain managers responsible for high-value items would benefit from the implementation of a blockchain supply chain ledger. High-value items that hospitals must track include organs for transplantation, blood products, expensive medications, operating room equipment and medical implants such as heart valves, and prosthetic blood vessels or hardware. Furthermore, some of these items, such as organs, blood products, and medications, may have stringent transport requirements such as transport time, temperature for transport, or regulatory transport compliance policies, which can be tracked in a distributed blockchain log. Blockchain’s traceability and transparency through the digital ledger can create a more efficient and effective way to manage high-value items along the supply chain. The distributed database will allow each party to come into contact with the high-value item to verify its location, compliance with transport requirements, and document handoffs. This will create transparency of transactions and the irreversible records entered in a distributed supply chain database [12]. This enables high-value items to be tracked in real time, improving inventory management, minimizing courier costs, identifying issues faster along the supply chain, reducing errors, and improving the use of limited resources such as operating room time [19]. Implementing blockchain technologies in the supply chain management of organ transportation should increase the rates of patients receiving allocated organs in a timely manner, and will provide a ledger that cannot be changed. By managing organ transportation from the donor hospital to the recipient with a distributed infrastructure, time sensitivity issues are monitored such that each transport team knows exactly when an organ is being removed, how long transportation will take, and how long after retrieval the organ will arrive at the recipient facility and placed into the transplant recipient. With electronic tracking, we can also monitor which parts of the organ transport system failed or lacked efficiency and work on improving the process. By using blockchain to track the organ in its journey from the donor to recipient, it can improve efficiency throughout the entirety of the process, ensure compliance with regulatory policies, and allow for innovation in organ transport, such as the use of drones for organ delivery.
2. One example of the potential use of blockchain in the health care industry is DonorNet, a secure internet-based system in which organ procurement coordinators (OPCs) send out offers of donated organs to transplant centers with compatible candidates. This system allows organ procurement organizations (OPOs) to add or modify information on donors and donor organs, initiate the donor-recipient matching process, and record organ placement information once the organ is accepted by the transplanting center based on the United Network of Organ Sharing (UNOS) designated policies. The donor-recipient match process ranks all matched, active candidates with specific information entered for a given donor organ. The resulting match list is the guideline by which individual organs are offered to listed transplant candidates.
3. OPO personnel post donor information in an electronic file format for review by transplant personnel. Such files include the OPO’s donor information form, ancillary confirmatory information such as ABO blood type, past social history, medical history, consent for donation, serology results for communicable diseases, digitized x-ray images, and other UNOS-mandated information regarding the donor. The intent of DonorNet was to remove an intermediary for organ acceptance and communicate directly with the physician or surgeon making the organ acceptance decision. By viewing posted source documents in a UNOS-specified and consistent fashion in DonorNet, transplant center personnel can reach an informed decision of whether to accept the organ for their transplant candidate [25,26]. Updating information into DonorNet is not a static process; however, currently, there is no way to notify the potential transplant centers of updated donor information other than the OPC reaching out with a phone call. Blockchain technology would avoid the time delay for a phone call and put in a system of checks and balances such that each party in the organ offer transaction acknowledges updated information, which is an important patient safety issue.
4. Once the organ is accepted by the transplant center, for example, kidney allografts, the retrieval process may occur long distances away from the transplant center. Currently, organs are shipped via transport companies, including commercial airlines and commercial courier services in some circumstances. With the implementation of blockchain technology, organs could be tracked more precisely along their transport route with a recording of each point of data entry and physical contact along with any important information about the condition of the organ during transport (such as pump perfusion numbers). According to the adoption framework being used [12], applications such as tracking access to EHRs that have a high degree of novelty and complexity or coordination will have slow adoption. Applications such as transplant organ availability and transport tracking of these organs are less innovative and require much less coordination with current EHR infrastructure, implying faster adoption. These functions allow for our organ transport example to keep patient information private and secure; however, it allows the transplant center to allocate limited resources, such as operating room time, in a more efficient manner. In our example, the organ and blood type may remain in the public domain for all users to know which organ is being tracked. However, PHIs such as the donor or the recipient may be protected under encryption and only those with a private key will have access to that data.
5. The next branch of the tree, medical devices, including implanted medical device technology, fall under the same branch as wearable medical devices. Implanted technology includes cardiovascular defibrillators, pacemakers, heart valves, and infusion pumps that must be surgically embedded into a patient. For example, in 2017, 465,000 pacemakers in the United States, and an additional 280,000 pacemakers internationally, were recalled by Abbott because of a design flaw in which hackers could access and *modify programming commands to the implanted pacemaker, which could result in patient harm from rapid battery depletion or administration of inappropriate pacing* [27]. The results of this hacking could lead to serious medical events or death. This example demonstrates where the same technology that allows for medical devices to be tracked along the supply chain can provide enhanced security for implanted medical devices. The security provided through the public or private keys, which allow verification to add new blocks or alter blocks to the chain, is valuable through the cryptographic hash function. Wearable medical devices and data from these devices are also included as part of health care. Wearable technology that can be improved through blockchain includes oxygen tanks, glucose monitors, and heart monitors. Implanted technology, including cardiovascular defibrillators, pacemakers, and infusion pumps, must be surgically embedded into a patient. For example, in the case of a glucose monitor, the patient or provider can issue a private key that *could automatically and securely record a patient’s blood glucose levels, and then, potentially, communicate with an insulin delivery device to maintain blood glucose at a healthy level* [28]. This tracking of wearable medical devices would allow for information to be tracked by patients and providers in real time.

Finally, the last branch under medical devices is high-tech medical machines. High-tech machines in hospitals include the da Vinci Surgical System, NvisionVLE Imaging System for Advanced optical coherence tomography imaging, and even atom-smashing machines used in cancer treatment. Similar to high-value medical items from the other branch of this tree, tracking these items along the supply chain is important for the machine’s maintenance and performance logs. Applying blockchain technology to the maintenance and performance logs would require any person logging in, such as a provider completing a procedure or an engineer completing maintenance, to log in with their private key. A record would be provided for every use, update, or repair performed on these expensive machines while clearly tracking the machines’ use throughout their lifecycle.

Blockchain has the ability to affect organ transplants not just in supply chain management but also in smart contracts and data exchange. Blockchain acts as a replacement for paper records and allows for a real-time view of each transaction along the supply chain process. As there is no central authority, all users have the authority to add transactions, but they may not change any history. This provides transparency in the process and makes blockchain a safe, interoperable solution to organ transplant and other medical technologies [29].

## Predictions for Blockchain Technology

**Figure 5:**
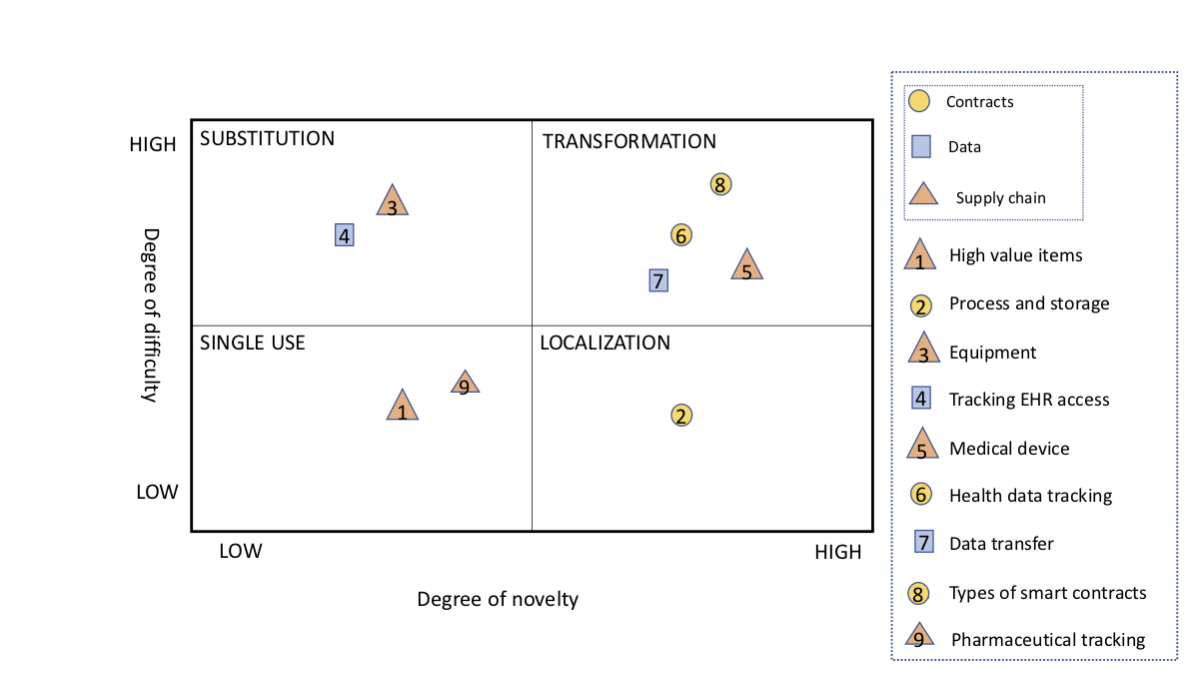
Evaluation of the proposed applications using Marco’s framework. EHR: electronic health data.

Figure 5 illustrates the use of the framework by Iansiti to display the degree of novelty and complexity of each proposed application and the speed of likely adoption. The first quadrant is single use. These technologies are *low-novelty and low-coordination applications that create better, less costly, highly focused solutions* [12]. The location of each mark in the quadrant indicates the level of novelty and complexity. Email is a non–health care single-use example, which provides an inexpensive alternative to traditional mail. Blockchain technology in its beginnings fell into this quadrant when it was being utilized only as cryptocurrency. In the health care field, high-value items supply chain management fits into the single-use category. This quadrant would be the easiest and quickest to adapt into health care organizations because it is low novelty and requires low levels of coordination and complexity to implement. One example of application in this quadrant is tracking high-value items such as organs, blood, and IV fluids. The ability to trace real-time movement along the supply chain provides unprecedented traceability and transparency for organizations. Another example is pharmaceutical drug tracking. Recently, SAP and Chronicled teamed up to produce a blockchain solution to detect counterfeit drugs. The application would allow multi-user verification to track prescription drug routing within the SAP collaboration hub [30]. The adoption of a blockchain would allow wholesalers and pharmaceutical companies to increase information sharing across the industry to improve patient safety. The low levels of coordination, complexity, and novelty make these technologies contenders for the first blockchain technology to be implemented in health care. The ease of implementation, transparency, accountability, and efficiency would be immediately beneficial for the installation of these technologies.

Although blockchain is not a novel concept, the use of blockchain itself may not be necessary in all of health care. Health care values privacy, transparency, and integrity more than the anonymity of user input [31]. Therefore, utilizing parts of blockchain technology, such as tamper-resistant technology, may increase the security of patient data and supply chain of high-cost necessities.

The next quadrant is localization, which *comprises innovations that are relatively high in novelty but need a limited number of users to create immediate value; therefore, it is still relatively easy to promote their adoption* [12]. This indicates that health care organizations would build on their single-use quadrant to branch out into implementing other blockchain-based technologies. Building of the single use of a high-value item supply chain would be the localization of process and storage. This would be the implementation of various technologies under the data exchange and contract trees, which would allow for blockchain technologies to begin storing data and processes on a blockchain network. For example, the storage of signed smart contracts or records of those who have accessed patients’ EHRs.

The third quadrant is substitution, which is the predicted utilization of blockchain that will become compelling substitutes or replacements of existing technologies. This technology can only be implemented by building off the foundational technology implemented in the single-use and localization quadrants. This technology is “relatively low in novelty because they build on existing single-use and localized applications... These innovations aim to replace entire ways of doing business.” An example of this would be the different cryptocurrencies that have evolved out of Bitcoin. In this quadrant for the health care industry, there is the equipment supply chain or the implantable and wearable medical devices, tracking EHR access, and supply chain to improve current issues of cost and transparency. However, this technology can only be implemented by building on the foundational technology implemented in the single-use and localization quadrants.

The last quadrant is transformation, which, like substitution, is high in its degree of coordination and complexity to implement and high in its degree of novelty. This technology *fundamentally changes the way businesses are created and capture value* [12]. Industry leaders use these technologies as keystones that proactively organize, influence, and coordinate the spread of networks of communities, users, and organizations [12]. This technology can be used for various types of smart contracts, health data tracking, the medical device supply chain, and data exchange. Smart contracts have the power to transform the system as they automate payments once the negotiated conditions are met in addition to being completely transparent and accessible. Health tracking data and data exchange will allow for better analytics to occur that will be easier for patients to understand and allow for bigger data to be more easily manipulated for better understanding. The items under transformation are long-term goals, whereas single-use application and localization applications can be achieved in the near future.

## Example of an Organ Transport Database and Transpiration Tracking

There is no lack of blockchain utilization in the health care and supply chain. One major area of interest for blockchain technology that has not been thoroughly investigated in health care is organ transplant supply chain management. The distributed blockchain ledger will record any individual who has come in contact with the organ as well as their location. Individual transactions are added into a block and cannot be altered. This system to record transportation information will not only help track organs but also distribute them to the appropriate locations within an allotted amount of time. All users who have access to a cryptographic key are able to track each step of their transportation. This ability is because each network user has their own replicated copy that allows for accountability and inhibits information tampering. Unlike in other industries, if 1 step of the supply chain process is delayed or halted, a recipient may lose their opportunity to be transplanted because organ transportation is highly time sensitive. Implementing blockchain technology and adding time alerts may remove the opportunity for error from the process.

Overall, the lack of novelty and complexity makes supply chain management in organ transplant a viable and feasible solution. Organ transport tracking via blockchain is feasible because it is easily adopted owing to its low novelty and complexity, as shown in Figure 5. Supply chain management of high-value items is common in health care and other industries. As discussed in the framework, using blockchain in supply chain management for high-value items is not a novel idea. Industries such as fishing and farming have been using this technology for years with great success. Implementing the technology in health care should be a relatively smooth transition as many flaws from lessons learned by the other industries have been discussed in this paper.

Startup technology companies have already begun experimenting with using blockchain in pharmaceutical supply chain tracking. Blockchain technologies have helped optimize the manufacturing, distribution, and dispensing of pharmaceutical products. They found that managing the supply chain *end-to-end* has decreased the amount of counterfeit medicines used for patients [32]. Another advantage of using blockchain technology in pharmaceuticals is that it can manage drug recalls easily by locating exactly who had access to the drug in question. The ability to use smart contracts also helps automate processes to reduce costs.

The complexity of developing the blockchain codes is simple and user friendly once implemented. Individuals provided with the key to enter the blockchain will be able to identify organs and their locations. This technology will increase the accountability of all involved personnel, from the procurement of the organ to the final delivery of the organ. Another factor in reducing the complexity of implementation of the supply chain management of transport organs is that this process is likely not part of the organization’s EHR. This independency from EHRs and its vendor makes the adoption of this application simple.

As discussed throughout the paper, tracking transport organs is likely for fast adoption because of its common use in supply chain management, independence from the EHR, and advantages to the current infrastructure. The implementation of blockchain will facilitate communication in real time, identify any issues during transport, and allow for better efficiency in the use of limited resources (eg, hospital beds for admissions or time). The implementation of blockchain to supply chains in health care can help track each step of the transportation process from procurement of the organ to the surgery of the recipient.

### Conclusions

The transformative power of blockchain has proven beneficial in non–health care industries. Blockchain has benefited non–health care industries with improved supply chains in terms of accountability, traceability, and transparency. It has proven its ability to improve outdated methods in other industries and can be applied to the health care industry as well. Application to health care will first be as single use in high-value items’ supply chain, but we predict that it will eventually reach transformation and disrupt the industry through innovations such as smart contracts. Enabling blockchain to solve many complex issues that the health care industry encounters today will allow a transformation that will lead to improved and innovative methods for viewing the health care industry. Although we discussed many uses of blockchain in this paper, we do not suggest using blockchain in its current form. Depending on the needs of a company, the DLT can be adapted from traditional blockchain uses to suit its needs.

## Appendix

Multimedia Appendix 1 Blockchain in other industries.

## Abbreviations

DLT: distributed ledger technology
EHR: electronic health record
FHIR: Fast Healthcare Interoperability Resources
IoT: Internet of Things
IT: information technology
IV: intravenous
OPC: organ procurement coordinator
OPO: organ procurement organization
PHI: personal health information
