# Supplementary material for: Adoption of Blockchain in Health Care
Source: J Med Internet Res. 2020 Sep 17;22(9):e17423. doi: 10.2196/17423 (PMC7530694; doi:10.2196/17423)
Supplement: Multimedia Appendix 1 [file jmir_v22i9e17423_app1.docx]

**Appendix I**

**Blockchain in Other Industries**

*Farming and Fishing Industry*

The fishing and farming industry have successfully employed Blockchain technology. Specifically, the power of Blockchain’s ability to provide supply chain information creating a positive impact on these industries. The nature of the fishing and farming industry is an international, multi-geographical, and multi-stakeholder complex series of relationships. Part of this complexity is due to lack of transparency and communication in current supply chain methodologies; however, Blockchain CAN solve these issues.

Due to Blockchain’s traceability and transparency, it works as a shared, secured digital ledger. This digital ledger has many applications that are useful in supply chain such as a distributed database where each party verifies the records directly, peer-to-peer transmission through a central node, transparency of transactions, and the irreversibility of records once a transaction is entered into the database. [12] These attributes of Blockchain are useful in supply chain due to their applications for contacts, tracking of goods, and record of payment in real time. [28] This, in turn, will provide the following benefits for the stakeholders involved in supply chain transactions: improvement of inventory management, minimization of courier costs, reduced delays from paperwork, faster identification of issues, increased trust between stakeholders, and the reduction or elimination of fraud and errors. [19]

Currently, non-healthcare organizations have already successfully employed Blockchain technology in their supply chain. For example, Provenance is an organization that provides Blockchain supply chain technology that has been successfully utilized in the tuna industry. Blockchain technology is utilized on mobile phones through smart tagging to track yellowfin and skipjack tuna in Indonesia. This verified social sustainability claims and provided evidence of compliance at origin and across the supply chain in a robust and accessible format for centralized data management system in the fishing industry. [3] This was achieved along with smart tags to track physical products from origin to point of sale through peer-to-peer technology from fisherman using a mobile smartphone application to consumers being able to track their fish from origin to supermarket.

Furthermore, Provenance has successfully utilized supply chain Blockchain in the coffee, coconut, and general farming markets. In the coffee industry, Provenance is utilizing data verification through supply chain for coffee producers to verify the source of their coffee beans. [4] The coconut industry includes an international supply chain, and in this case Blockchain supply chain has been utilized to ensure that the 55 farmers are being paid a fair living wage from their employer to track ethical claims and ensure fair trading practices. In conjunction with the NGO Fairfood, supply chain Blockchain was tracked harvesting via SMS and payment through verification of custody along the supply chain. Finally, Provenance has proven Blockchain supply chain power through the organic food industry in the United Kingdom. Working with the Soil Association organic certification process, Provenance created an interactive certification that allows consumers to screen the certification and see the product’s path from origin to store. This allows for a transparent supply chains for the industry to track their products as well as consumers. [5]

*Textile and Fashion Industry*

The textile and fashion industry have used Blockchain technology to increase transparency through a peer-to-peer database that is transparent and secure. Similar to the fishing and farming industry, Blockchain supply chain technology can be utilized to track textiles such as cotton. The Seam is a global agri-business and commodities trading software provider that formed a consortium with IBM to transform the global cotton industry. The Seam lead an “industry-wide collaboration initiative to create a supply chain and trading ecosystem built on IBM Blockchain technology.” [6] Utilizing the shared, secured digital leader and smart contacts, The Seam and IBM aim to create greater efficiency and global collaboration in the industry.

London based Martine Jarlgaard worked with Provenance to enable Jargaard to verify information about the textiles acquired for their clothing. Tracking the raw material (alpaca fleece) from shearing on the farm through the spinning and knitting process eventually to Jarlgaard’s London studio. This allowed for a unique ID to be added into the label which allowed for location mapping, content, and timestamps for each part of production from shearing to purchased garment. [7]

*Diamond Industry*

The diamond industry has also utilized Blockchain technology for the purpose of tracking items through complex supply chains. Miners in the industry along with consumers want to have the ability to trace gems to their origination as well as their destination. [12]

De Beers, the world’s biggest diamond producer has taken the initiative to turn to Blockchain technology to track gems from miner to retailer. [8] They want to guarantee the ethical source and maintain consumer confidence. De Beers is running a pilot study to understand Blockchain’s potential use for supply chain as is a London-based company, Everledger, which aims to launch the first industry-wide Blockchain to track gems from the moment they are dug from the ground to every time they change locations. [8] Blockchain technology “will enable De Beers to show transactions to all participants while keeping their identities and the value of the sales hidden,” [8] which will significantly help eliminate the entry of fakes and conflict stones that have previously hurt the diamonds’ reputation and contributed to lower prices [8].

With the use of Blockchain technology, Everledger, has successfully placed more than 1.6 million diamonds on a Blockchain. “Entries of the digital record include dozens of attributes for each diamond, including the color, carat, and certificate number, which can be inscribed by laser on the crown or girdle of the stone.” [9] The purpose of the technology is to replace paper certification with the Blockchain ledger. Computer scanning tools will be used to verify the authenticity of the gems. The use of Blockchain platform will enable all participants from consumers to dealers to have confidence in the diamonds. Diamond Blockchain benefits include permanence and assurance. Once a diamond is digital added, it cannot be altered, which helps ensure the diamond’s attributes. It is a secure platform to share confidential information and lessens the need of manual handling for an efficient, streamlined process.

*Floral Industry/Shipping*

A shipping company known as Maersk FAMOUS FIRM MARK has been working with IBM to use Blockchain to track shipments across seas. [10] The technology will enable the company to not only track the cargo containing the contents but also the individual contents of the boxes. It will eliminate the use of paperwork for global supply chain shipments with tamper-resistant digital records. [11] A pilot study was conducted to trace a container of flowers to test the system’s potential for cargo movement and global trade. The opportunities include increasing efficiency and timeliness for cargo movement, which will reduce lost or delay in goods, especially perishable goods to avoid spoilage and costly replacement of wasted products. As perishable and nonperishable goods are shipped globally, the use of Blockchain technology will ultimately enhance health, safety and regulatory benefits across multiple industries.
